# The Predictive Role of Cognitive Emotion Regulation of Adolescents with Chronic Disease and Their Parents in Adolescents’ Quality of Life: A Pilot Study

**DOI:** 10.3390/ijerph192316077

**Published:** 2022-12-01

**Authors:** Melinda Cserép, Brigitta Szabó, Péter Tóth-Heyn, Attila J. Szabo, Irena Szumska

**Affiliations:** 1 Institute of Behavioural Sciences, Semmelweis University, 1089 Budapest, Hungary; 2 First Department of Pediatrics, Semmelweis University, 1083 Budapest, Hungary; 3 Doctoral School of Psychology, ELTE Eötvös Loránd University, 1064 Budapest, Hungary; 4 Institute of Psychology, ELTE Eötvös Loránd University, 1064 Budapest, Hungary

**Keywords:** chronic illness, inflammatory bowel disease, type 1 diabetes, quality of life, cognitive emotion regulation, self-blame

## Abstract

Background: The purpose of this study was to investigate cognitive emotion regulation in adolescents with chronic illness and their parents. Methods: Eighty-five young people (mean = 15.86 years, standard deviation = ± 1.42, girls 65.88%) with chronic illnesses (inflammatory bowel disease n = 40 or type 1 diabetes n = 45), and their parents (mean = 46.06 years, 87.06% mother) completed the Cognitive Emotion Regulation Questionnaire (CERQ) for themselves and the Inventory of Quality of Life in Children and Adolescents (ILC) questionnaire adolescent and parent version. We conducted two hierarchical linear regression analyses with “enter” method. The CERQ scales and the diagnosis of chronic disease were chosen as independent variables, and the total ILC score in the first analysis and the ILC proxy score in the second analysis were chosen as dependent variables. Results: Among adolescents, cognitive emotion regulation strategies such as self-blame, positive reappraisal, and catastrophizing have been proven to be predictors of their own quality of life; however, parental self-blame was also found to be a predictor of adolescents’ quality of life. Parental rumination and positive refocusing have been shown to be predictors of how parents rate their child’s quality of life. Conclusions: The present study sheds light on cognitive emotion regulation strategies in adolescents with chronic illness and their parents that have a significant impact on the development of young people’s quality of life.

## 1. Introduction

A multidisciplinary approach to the treatment of people with chronic diseases, including the improvement of their quality of life, has become a major focus of treatment. As well as improving the physical and mental well-being of individuals, better mental well-being and quality of life can also help to reduce healthcare spending on treatment [1,2,3,4]. A wide range of research is underway with the aim of developing an improved understanding of psychological predictors of quality of life among chronic patients [5,6]. The role of cognitive emotion regulation is a particularly promising area that holds therapeutic potential for clinical practice [7].

Adaptive regulation of emotions, which allows individuals to better control and manage emotional experiences or current situations is key to everyday adaptation to environmental factors [8]. In adolescence, emotional ups and downs and difficulties in regulating more negative moods are developmentally specific [9,10]. On the one hand, adolescence is a common period of emergence for many psychopathologies, and on the other hand, it is a very important phase for the development of emotion regulation through changes in cognitive development [9,11], which optimally contributes to more adaptive coping mechanisms in adulthood. Young people with chronic illness have more difficult developmental tasks compared to their healthy peers; for them, independence and separation from their parents are inextricably linked to their illness. Such adolescents often develop a very close relationship with the mother, who usually manages tasks related to the illness, while the father is less present in the life of the family [12,13,14]. These characteristics of chronic illness can result in additional problems (for example, fear of failure and anxiety about family and social expectations), which can lead to the development of psychological problems. Higher risks of depression and anxiety and other mental health problems have been described in several studies in young people with chronic illness [15,16,17,18] and individuals’ mental state is often associated with adherence problems and worsening of physical symptoms [19,20]. Current research results suggest that problems with emotion regulation are transdiagnostic factors in the development of mental illness, and therefore adaptive regulation of negative emotions is key to maintaining mental health [7,8,21,22,23,24,25,26].

Research on the relationship between emotion regulation and chronic illness highlights that non-adaptive emotion regulation impairs adaptation to chronic illness and increases the likelihood of developing mental health problems [20,27]. In the adult population, Tombini and colleagues [28] investigated emotion regulation, depressive symptoms and quality of life in epilepsy patients. Their results show a two-way relationship between depressive symptoms and emotional dysregulation, and higher levels of both are significantly associated with a poorer quality of life. Bahrami and colleagues [29] investigated emotion regulation in adults with cancer, where positive refocusing, positive reframing and putting into perspective were significantly positively related to quality of life, while other-blame was significantly negatively related. In their study, the only predictor was positive reframing. Examining the cognitive and behavioral coping behavior of adolescents with type 1 diabetes (T1DM), Kraaij and colleagues [30] described several cognitive emotion regulation strategies related to depression (rumination, catastrophizing, self-blame, blaming others, acceptance, positive refocusing and positive reappraisal), while behavioral coping strategies were not significantly associated with emotion regulation strategies. The authors highlight the importance of developing cognitive strategies in adolescents diagnosed with T1DM.

Bogdan and Gherasim-Diaconu [31] examined adolescents with cancer and found cognitive emotion regulation strategies to be predictors of mental well-being as well as adaptation to the disease. Positive reframing predicted psychological well-being, while ruminating and blaming others predicted adjustment to illness. Garnefski and colleagues [32] found catastrophizing and rumination to be the most predictive of quality of life in adolescents diagnosed with juvenile idiopathic arthritis. Kraaij and Garnefski [33], in their multidisciplinary research on chronic illness, found cognitive emotion regulation strategies to be of paramount importance in depression, highlighting the role of self-blame, rumination and catastrophizing. Reed and colleagues [34] highlight the importance of catastrophizing when examining the quality of life of adolescents with inflammatory bowel disease (IBD).

Parental emotion regulation plays an important role in the development of children’s emotion regulation. Difficulty in parental self-regulation reduces the parent’s emotional availability and responsiveness to their child [35]. Morris and colleagues [36] developed a three-tier model that describes three interrelated processes in the development of emotion regulation that have a jointly decisive influence: observing model following and parental emotion regulation, parental and caretaker behavior and the emotional climate of the family, including parenting styles. The authors emphasize that this is a two-way process, in which the child also affects the parents, by their temperament and inherited characteristics.

While the development of emotion regulation in the early years of life is a thoroughly researched area in terms of the parent–child relationship [37,38,39], there are few studies [40] on the correlations of emotion regulation between parents and adolescents. Bariola [37] and colleagues investigated the relationship between the emotion regulation of children and adolescents aged 9-19 and their parents. Based on their results, mothers’ expressive suppression proved to be predictive of their children’s suppression strategy in the adolescent age group. This topic is particularly relevant for adolescents with chronic illness and their parents, where in many cases the process of separation begins later, and peer relationships are often poorer [41]. Parents caring for a chronically ill child must also manage their child’s physical and emotional well-being, looking beyond their own feelings about the disease, which often poses a significant burden [42]. Van Gampelaere and colleagues [43] found a significant association between maternal illness-related parenting stress and the psychological functioning of children with T1DM, but maternal adaptive strategies were not proven to be buffering.

Investigating cognitive emotion regulation strategies is thus a critical next step to identify important intervention points that can be applied among chronically ill patients at risk of developing psychological problems. Through the modulating effect of conscious attention and thinking processes, cognitive emotion regulation allows individuals more control over managing a given emotional experience or situation, and thus can be effectively developed through therapeutic methods [44,45]. The novelty of the present study is that we analyzed the role of parents’ and adolescents’ emotion regulation alike.

In our research, we examined the cognitive emotion regulation of young people with chronic illnesses (T1DM, IBD) and their parents in the context of quality of life. Both examined diseases are lifelong but affect patients in different ways. While the lives of adolescents living with type 1 diabetes are dominated by attention to diet, blood sugar measurement and insulin dosing [46,47,48], young people with IBD must cope with the fluctuating course of the disease and gastrointestinal symptoms [49,50]. T1DM and IBD have several similar psychosocial impacts on adolescents, including depression, anxiety, disordered eating, independence issues, etc. [13,51,52]. The objective of our study was not to focus on somatic factors specific to each disease but to assess the cognitive emotion regulation strategies that play a role in the quality of life of young people. We consider the examination of cognitive emotion regulation strategies to be highly promising because it provides an opportunity to assess adaptive and non-adaptive strategies in detail, and as a transdiagnostic factor, it offers a very broad scope for prevention and intervention. In our research, we aimed to identify adaptive and non-adaptive strategies whose development can contribute to a better quality of life for young people and a smoother adaptation to disease. 

The following hypotheses were investigated: (1) adolescents’ quality of life is predicted by the adolescents’ emotion regulation strategies and their parents’ negative emotion regulation strategies; (2) both parents’ and adolescents’ emotion regulation strategies are predictive of proxy quality of life; (3) the cognitive emotion regulation strategies catastrophizing and rumination are the main predictors of quality of life, based on previous research on adolescent chronically ill patients [32,34,53]; (4) rumination is the strongest predictor of proxy quality of life [53].

## 2. Materials and Methods

### 2.1. Participants

The study included 170 subjects, of whom 85 were Caucasian adolescents with chronic illness between the ages of 14 and 18 (*M* = 15.86 years, *SD* = 1.42) and 85 were parents of the participating adolescents. The inclusion criteria for participants included adolescents: (1) with a diagnosis of T1DM or IBD and one of their accompanying parents; (2) diagnosed with the disease at least six months ago; (3) aged 14 to 18 years; (4) gave written informed consent together with their parents. (Exclusion criteria were: (1) unaccompanied youth; (2) intellectual disability).

Forty (47.1%) adolescents had a diagnosis of inflammatory bowel disease (IBD) and forty-five (52.9%) had type 1 diabetes (T1DM). Among the patients completing the questionnaire were 56 girls (65.9%) and 29 boys (34.1%). The majority of parents were mothers (*n* = 74, 87.1%), while 11 fathers (12.9%) participated in our study. The demographic data are summarized in Table 1.

### 2.2. Procedures

The subjects recruited were adolescents from 14 to 18 years of age with IBD and T1DM diagnoses who received outpatient or inpatient care at the First Department of Pediatrics, Semmelweis University (Hungary) and their accompanying parents. A paper questionnaire package was completed. There were four incomplete questionnaire packages in total. Eleven individuals chose not to participate in the study. The completion of the questionnaire was carried out with written informed consent from both parents and adolescents. We provided accurate information on the purpose of participation and the research. Completion was completely voluntary and anonymous. Participants did not receive any financial incentive for completing the questionnaire. 

### 2.3. Measures

The Cognitive Emotion Regulation Questionnaire (CERQ) was developed by Garnefski [45]. The questionnaire examines cognitive-emotional regulation strategies for stressful life events. The 36-item questionnaire allows for the identification of nine types of emotion regulation mechanisms, five of which are adaptive (positive refocusing, planning, positive reappraisal, putting into perspective, acceptance), four non-adaptive (self-blame, other-blame, rumination, catastrophizing). There are four items in the questionnaire assessing each cognitive emotional regulation strategy, rated by the subject on a 5-point scale from 1 ((almost) never) to 5 ((almost) always). A higher score indicates more frequent use of the cognitive strategy. The CERQ questionnaire has good psychometric indicators on both adolescent and adult samples [45]. In the Hungarian sample [54], CERQ also proved to be a reliable measuring tool. In the present study, CERQ also had good internal consistency across all scales of CERQ; Cronbach’s alpha was 0.88 in the case of adolescents and 0.83 in the case of parents. In our study, the reliability indices of the adolescent self-administered questionnaire, the Cronbach’s alpha values, varied between 0.63 and 0.88, the former on the self-blame and the latter on the positive refocusing scales. For parents, Cronbach’s alpha values ranged from 0.62 to 0.89, the former on the acceptance and catastrophizing scales, and the latter on the positive refocusing scales. 

The Invertar Lebensqualität Kindern und Jugendlichen (Inventory of Quality of Life, ILC) is a 7-item quality of life questionnaire [55]. The questionnaire asks about the main areas of life of children between the ages of 6 and 18, with different versions for children, adolescents and parents The latter is parents’ perception of their child’s quality of life. We used the adolescent (ILC) and the parent (ILC proxy) versions. The questionnaire is answered on a 5-point scale, where 1 is the best and 5 is the worst quality of life. The total score is calculated by adding up the scores from 1 to 5 and subtracting this sum from 35, so possible scores fall between 0 and 28. A higher score indicates a better quality of life, and a lower score a worse quality of life. ILC was validated in the average German population and in a psychiatric sample, with internal consistency ranging from 0.55 to 0.63 for children and adolescents, and Cronbach’s alpha ranging from 0.66 to 0.76 for parents [55]. Studies performed on a sample in Hungary [56] verified the questionnaire had adequate reliability and validity. In our study, both the adolescent ILC and the parental version had good internal consistency (Cronbach’s alpha: 0.83 and 0.81, respectively).

### 2.4. Statistical Analyses 

IBM SPSS Statistics (Version 27) was used for the analyses. The skewness and kurtosis of the scales were checked. If it was acceptable (between ±1) [57], then we used parametric tests. Bonferroni correction was applied to consider multiple testing (significance level: α/n; n = number of analyses). To demographically compare groups with IBD and T1DM diagnoses, chi-square tests were used for categorical variables, and independent samples t-tests were used for continuous variables and appropriate skewness and kurtosis, and Mann–Whitney U tests were used for continuous variables but inappropriate skewness and kurtosis. The difference in scores on the scales per diagnosis was checked by an independent samples t-test, while in the CERQ questionnaire, the other-blame subscales were transformed for both parents and adolescents, and then an independent samples t-test was used for comparison by diagnosis. The group differences between demographic variables and the scales used were examined using independent samples t-tests and one-way analysis of variances (ANOVA). The correlation between the CERQ and ILC scales was examined by Pearson correlation. We conducted two hierarchical linear regression analyses with the “enter” method. We chose predictors for the regression analyses based on significant Pearson’s correlation coefficients (*p* < 0.0025); furthermore, we controlled the regression analysis for the diagnosis by adding it in the first step. Multicollinearity was controlled by means of tolerance (TOL < 0.10) and variance inflation factor (VIF > 10). On the basis of these criteria, none of the analyzed variables showed multicollinearity. 

## 3. Results

### 3.1. Descriptive Statistics and Preliminary Analyses 

The descriptive characteristics of the CERQ and ILC scales are illustrated in Table 2 in detail.

There was no significant relationship between demographic variables and scales. Since our study included adolescents diagnosed with two different chronic diseases, we examined whether there were any differences in demographic variables (Table A1, Table A2 and Table A3) and CERQ and ILC scales (Table A4 and Table A5) between adolescents diagnosed with IBD and T1DM and between parents of adolescents diagnosed with IBD and T1DM. A significant difference was found between the two groups regarding the variable duration of illness (how many years ago the disease was diagnosed), *t*(80.99) = −4.27, *p* < 0.001, *d* = 0.92, with adolescents with T1DM having been diagnosed earlier (*M* = 7.01, *SD* = 4.23) than adolescents with IBD (*M* = 3.56, *SD* = 3.19) (Table A1). Within the CERQ questionnaire, there was a significant difference in positive reappraisal, indicating that adolescents diagnosed with T1DM used more positive reappraisal than adolescents with IBD (Table A2). No further significant differences were found between the IBD and T1DM groups, so they were not separated in the following statistical analysis (Table A2, Table A3, Table A4 and Table A5).

### 3.2. Correlation of ILC and CERQ Scales

The adolescents’ ILC quality-of-life scores showed a moderate relationship with the following CERQ scales: self-blame, catastrophizing and positive reappraisal. Self-blame and catastrophizing were negatively correlated with quality of life, while positive reappraisal was positively correlated. Furthermore, ILC was significantly negatively correlated with parental self-blame, and the strength of the relationship was also moderate. The ILC proxy was significantly negatively correlated with parental CERQ rumination and self-blame, while it was positively correlated with proxy positive refocusing. The strength of these associations was also moderate. The correlation results for the scales are summarized in Table 3.

### 3.3. Hierarchical Linear Regression Analysis of Cognitive Emotion Regulation Strategies and Quality of Life

Hierarchical linear regression analysis was performed using the “enter” method to examine the predictive role of variables, which were found to be significantly correlated with the ILC score and ILC proxy score in the preliminary results. To analyze the predictive role of chronic disease, the diagnosis was also included among the independent variables.

#### 3.3.1. First Hierarchical Linear Regression Analysis

Adolescents’ quality of life was chosen as a dependent variable, and the diagnosis, CERQ self-blame, catastrophizing, positive reappraisal and parental CERQ self-blame were included in the analysis as independent variables using the “enter” method. Each of the independent variables was added in different steps. The results of the hierarchical linear regression analysis are shown in Table 4. The models were significantly improved by adding more predictors in each step, and the final model explained 49.4% of the variance in ILC. The diagnosis was a significant predictor till the third step. The T1DM group predicted a higher ILC score than the IBD group. However, in the fourth model, when self-blame, catastrophizing and positive reappraisal were also included in the analysis, the diagnosis was no longer a significant predictor of the ILC. Based on the standardized regression coefficients, self-blame was the strongest predictor, followed by positive reappraisal, catastrophizing and parental self-blame. Only positive reappraisal predicted a higher ILC score; the other predictors were associated with lower levels of ILC. 

#### 3.3.2. Second Hierarchical Linear Regression Analysis

Proxy adolescents’ quality of life (ILC proxy) was chosen as a dependent variable, and diagnosis, rumination, self-blame and positive refocusing were included in the analysis as independent variables using the “enter” method. Each of the independent variables was added in different steps. The results of the hierarchical linear regression analysis are shown in Table 5. The models were significant and were improved upon in each step with the addition of more predictors. However, the explained change in variance was not significant in the third model. The last model explained 32.9% of the variance in the ILC proxy. The diagnosis was a significant predictor in all the models; the T1DM group had a higher predicted ILC proxy score than the IBD group. Parental self-blame was not a significant predictor of the ILC proxy, while rumination predicted a lower ILC proxy level and positive refocusing predicted a higher ILC proxy. Based on the standardized regression coefficients, parental rumination was a stronger predictor of ILC proxy than the diagnosis. 

## 4. Discussion

Our study investigated the role of cognitive emotion regulation strategies of adolescents with IBD and T1DM and their parents on adolescents’ quality of life, using both self-report and proxy questionnaires. An interesting feature of the study was that the predictive role of cognitive emotion regulation strategies specific to adolescents and parents was analyzed together.

First, we analyzed the bivariate relationships. Three adolescent cognitive emotion regulation strategies were found to be significantly correlated with self-related quality of life: self-blame, catastrophizing and positive reappraisal. Our hypothesis was not confirmed, as rumination did not significantly correlate with quality of life in our study. An interesting finding was that among parents’ cognitive emotion regulation strategies, self-blame was correlated with adolescents’ quality of life. Parental rumination, self-blame and positive refocusing were related to parent proxy quality-of-life scores.

Although our research was not aimed at comparing the groups diagnosed with T1DM and IBD, diagnosis was included as an independent variable in both hierarchical linear regression analyses, as the sample was heterogeneous. Regarding the adolescents’ self-rated quality of life, the diagnosis proved to be a significant predictor on its own and after including two cognitive emotion regulation strategies; however, after positive re-framing and parental self-blame were included in models 4 and 5, the predictive role of diagnosis disappeared. In our study, adolescents diagnosed with T1DM had a significantly longer disease duration than IBD patients, and the T1DM group had a higher predicted ILC score. Previous research has reported [5,58,59] improved quality of life over time in people with chronic illnesses, which may be related to the process of adapting to the illness; this phenomenon may partially explain the results obtained for adolescents with T1DM. In terms of cognitive emotion regulation, positive reappraisal was significantly more prevalent in adolescents with T1DM. Further studies are needed to determine whether this difference is due to the nature of the two diseases, the longer disease duration or other factors.

In our study, three adolescent cognitive emotion regulation strategies were found to be predictive of the self-rated quality of life of adolescents with chronic illness based on the hierarchical linear regression analysis, thus partially confirming our hypothesis regarding the predictive role of rumination and catastrophizing. Interestingly self-blame was found to be the strongest predictor of young people’s quality of life, with positive reframing and catastrophizing also playing explanatory roles. The role of self-blame is being researched in lifestyle-related diseases, as well as in association with the onset of illness, where self-blame is a well-known and common phenomenon [60,61,62]. Voth and Sirois [63] investigated health-related self-blame among adult IBD patients. They found that self-blame was associated with avoidant coping and poor adjustment to illness. In our research, not only did adolescent self-blame play a role in young people’s quality of life but parental self-blame was also considered an explanatory variable, confirming our hypothesis that parental maladaptive strategies are significant predictors of adolescents’ quality of life. This finding calls for further research on the correlation of self-blame between young people and their parents. Self-compassion, on the other hand, has been shown to be a promising strategy for reducing self-blame in the case of chronically ill patients [64]. 

Among adaptive emotion regulation strategies, our research highlights the importance of positive reframing in quality of life. In adolescents with chronic illness, positive reframing emerged as a predictor of self-rated quality of life, both for self-rated and proxy quality of life. Van De Ven and colleagues [65], studying adolescents diagnosed with asthma, also described an association between positive reappraisal and improved quality of life. The role of emotion regulation strategies, including positive reframing, has also been examined in relation to post-traumatic growth. A two-year follow-up of adult breast cancer survivors showed that positive reframing was associated with a greater post-traumatic increase [66]. Positive refocusing, which refers to redirecting attention from unpleasant events to pleasant ones, emerged as a predictor of parents’ estimates of their own child’s quality of life. The development of positive emotion regulation strategies can improve the quality of life and the psychological support of young people affected by chronic diseases and their parents.

Our hypothesis regarding the emotion regulation strategy of rumination in adolescents with chronic illness was not confirmed. Interestingly, however, parental rumination emerged as the strongest negative predictor of proxy quality of life, confirming our hypothesis that parental cognitive emotion regulation strategies also play a role in parents’ perceptions of their children’s quality of life. Rumination refers to a repetitive and passive engagement with experienced distress, its cause and possible consequences [67]. Relevant research has demonstrated its link to a number of psychiatric illnesses, with most studies in this area being focused on its association with depression [68,69,70]. Fisher and colleagues [71] also emphasize the role of rumination. Their research shows that in parents of children with cancer, poorer parental perception of the child’s symptoms and pain increases parental distress through rumination. In a study by Kohut and colleagues [53], parents’ rumination on their child’s pain was found to be an important predictor of child quality of life in IBD patients. Our results suggest parental rumination is a very significant explanatory variable of the child’s proxy quality of life. Its clinical relevance also arises from the fact that a constant, unproductive preoccupation with thoughts and feelings about events that have occurred reduces parents’ ability to effectively problem solve and their capability to remain emotionally attuned to their child. Previous research has described a negative relationship between parental self-efficacy and rumination in parents of children with developmental disabilities [72]. In a sample of mothers of non-clinical children, self-blame mediated the relationship between low parental self-efficacy and parental role satisfaction [73]. The influence of the parent’s mental health on the child’s emotional well-being and quality of life has been the subject of several studies [5,74]. A way forward in terms of adaptation to chronic illness could be the introduction of group therapy for young people and their parents, focused on the development of emotion regulation as part of the mental health support toolkit.

## 5. Limitations, Strengths, and Implications for Research

It is necessary to mention some limitations of our research. This was a cross-sectional study, and thus not suitable for causal inference. It would be worthwhile to longitudinally investigate the association of cognitive emotion regulation strategies with changes in chronic disease symptoms and quality of life. Our sample size was small, so the results should be interpreted with caution, as further studies with a larger number of elements are needed to confirm the results. Additionally, there was a higher proportion of mothers among the parents who completed the questionnaire. A larger number of sample elements would also provide an opportunity to analyze differences between mothers’ and fathers’ emotion regulation strategies and to explore differences between disease groups in more depth. 

One of the strengths of our research is that we examined the cognitive emotion regulation of adolescents and their parents together. Our results—though they should be treated with caution—clearly point to the relevance of further research. The development of cognitive emotion regulation strategies can not only improve the quality of life but also reduce the overall chances of the development of psychopathologies and contribute to better mental health [5,74,75,76]. Our research shows links between highly relevant themes, such as coping behavior, self-efficacy, grit and resilience [77,78,79,80], and the development of healthcare management skills. These topics indicate a possible direction for more complex investigations in connection with cognitive emotion regulation.

## 6. Conclusions

The need and usefulness of measuring and developing cognitive emotion regulation strategies for chronic patients are clearly outlined in the studies cited and in our own research. Our study highlighted the predictive role of self-blame in the quality of life of adolescents with IBD and T1DM. Our findings also suggest that research and psychological support should also focus on parental self-blame. The parent–child relationship is an interdependent relationship that needs to be taken into account and utilized in clinical work. Our study points out that in the case of chronically ill adolescents, it is worth looking at parents’ and adolescents’ emotion regulation in parallel because both can affect the child’s quality of life.

A way forward in terms of adaptation to chronic illness could be the introduction of group therapy, involving young people and their parents, to help them develop emotion regulation strategies as part of the mental health support toolkit.

## Figures and Tables

**Table 1:** Demographic characteristics.

Characteristic	*M* (*SD*) or % (*n*)
Adolescent age (years)	15.86 (1.42)
Parent age (years)	46.06 (4.41)
Duration of illness (years)	5.39 (4.13)
Adolescent sex (female)	65.88 (56)
Parent sex (female)	87.06 (74)
Place of residence	
Capital city	27.06 (23)
City	52.94 (45)
Village	20.00 (17)
Education (highest of parents)	
College +	63.53 (54)
High school	22.35 (19)
Less than high school	14.12 (12)
Family	
Dual parent	80.00 (68)
Single parent	16.47 (14)
Other	3.53 (3)

Note. *n* = 85.

**Table 2:** Descriptive statistics and reliabilities of study variables.

	*M (SD)*	Min	Max	Skewness	Kurtosis	Cronbach’s α
ILC	20.17 (5.15)	8	28	–0.28	–0.66	0.83
CERQ	97.83 (18.92)	50.43	135	–0.31	–0.42	0.88
Self–blame	9.96 (4.11)	4	20	0.64	–0.30	0.86
Acceptance	13.10 (3.39)	6	20	–0.02	–0.49	0.63
Rumination	10.23 (4.25)	4	20	0.40	–0.57	0.81
Positive refocusing	11.24 (4.37)	4	20	0.15	–0.96	0.88
Planning	13.27 (3.61)	4	20	–0.32	–0.37	0.74
Positive reappraisal	12.49 (3.90)	4	20	0.01	–0.59	0.78
Putting into perspective	12.15 (4.23)	4	20	0.10	–0.87	0.84
Catastrophizing	8.23 (3.60)	4	17	0.72	–0.47	0.76
Blaming others	7.16 (3.24)	4	17	0.37	–0.97	0.86
ILC proxy	21.09 (4.11)	13	28	–0.29	–0.85	0.81
CERQ parent	96.31 (14.52)	59	139	–0.23	0.89	0.83
Self–blame parent	9.27 (3.37)	4	19	0.78	0.44	0.82
Acceptance parent	12.90 (3.13)	6	20	0.03	–0.27	0.62
Rumination parent	10.19 (3.27)	4	19	0.27	0.06	0.70
Positive refocusing parent	10.90 (3.83)	4	20	0.43	–0.53	0.89
Planning parent	14.96 (3.42)	4	20	–0.57	0.37	0.79
Positive reappraisal parent	13.20 (3.75)	5	20	–0.19	–0.58	0.78
Putting into perspective parent	12.03 (3.96)	4	20	0.24	–0.44	0.80
Catastrophizing parent	7.18 (2.57)	4	13	0.49	–0.79	0.62
Blaming Others parent	5.68 (18.8)	4	13	0.52	–0.95	0.72

Note. *n* = 85. ILC = Inventory of Quality of Life, CERQ = Cognitive Emotion Regulation Questionnaire.

**Table 3:** Bivariate relationships (Pearson’s correlation coefficients) of study variables (*n* = 85).

	1	2	3	4	5	6	7	8	9	10	11	12	13	14	15	16	17	18	19	20
1. ILC	—																			
2. ILC proxy	0.556 *	—																		
3. Self-blame	−0.500 *	−0.215	—																	
4. Acceptance	0.094	−0.036	0.275	—																
5. Rumination	−0.312	−0.238	0.543 *	0.277	—															
6. Positive refocusing	0.282	0.082	−0.157	0.313	0.055	—														
7. Planning	0.223	0.166	0.102	0.400 *	0.238	0.502 *	—													
8. Positive reappraisal	0.384 *	0.306	−0.037	0.350 *	0.133	0.639 *	0.573 *	—												
9. Putting into perspective	0.196	0.124	−0.008	0.381 *	0.095	0.532 *	0.391 *	0.582 *	—											
10. Catastrophizing	−0.452 *	−0.315	0.323	−0.004	0.365 *	−0.128	−0.056	−0.214	−0.077	—										
11. Other-blame	−0.118	−0.126	0.269	0.043	0.235	−0.058	0.083	0.049	−0.044	0.470*	—									
12. Self-blame parent	−0.337 *	−0.371 *	0.276	0.121	0.312	−0.053	−0.057	−0.019	0.045	0.144	0.138	—								
13. Acceptance parent	−0.007	0.171	0.104	−0.008	−0.255	−0.124	0.119	−0.031	−0.143	0.077	0.119	−0.134	—							
14. Rumination parent	−0.201	−0.472 *	0.250	0.002	0.297	−0.008	−0.043	−0.095	−0.016	0.225	0.140	0.569 *	−0.114	—						
15. Positive refocusing parent	0.075	0.334 *	−0.021	0.032	−0.112	0.021	−0.038	0.011	−0.010	0.175	0.038	−0.228	0.280	−0.305	—					
16. Planning parent	−0.016	0.022	0.234	0.127	0.105	0.001	0.151	0.102	0.142	0.188	0.236	0.092	0.138	0.289	0.289	—				
17. Positive reappraisal parent	−0.058	0.159	0.228	0.196	0.005	0.026	0.086	0.137	0.092	0.079	0.244	−0.096	0.221	−0.129	0.401*	0.548 *	—			
18. Putting into perspective parent	−0.032	0.150	0.181	0.183	0.027	0.077	0.138	0.196	0.087	0.147	0.125	−0.111	0.334 *	0.042	0.284	0.497 *	0.660 *	—		
19. Catastrophizing parent	−0.064	−0.278	0.023	−0.137	0.119	0.027	−0.011	0.053	−0.061	0.188	0.071	0.258	0.009	0.533 *	−0.209	0.116	−0.238	0.178	—	
20. Other-blame parent	−0.085	−0.186	0.263	0.070	0.324 *	−0.097	−0.038	−0.189	0.068	0.222	0.178	0.284	−0.328 *	0.387 *	−0.018	0.190	0.049	0.244	0.101	—

Note: * *p* < 0.0025 (α = 0.05/20, using Bonferroni correction), ILC = Inventory of Quality of Life.

**Table 4:** Hierarchical Linear Regression Analysis with ILC as Dependent Variable.

Model	*F*	df1	df2	*p*	R^2^	Change Statistics					Variable Added	B	Std. Error	β	*t*	*p*	Collinearity Statistics	
						R^2^	*F*	df1	df2	*p*							TOL	VIF
1	6.142	1	83	0.015	0.069	0.069	6.142	1	83	0.015	Constant	16.049	1.749		9.177	<0.001		
											Diagnosis	2.694	1.087	0.262	2.478	0.015	1.000	1.000
2	17.442	2	82	<0.001	0.298	0.230	26.830	1	82	<0.001	Constant	22.733	1.999		11.370	<0.001		
											Diagnosis	2.257	0.953	0.220	2.368	0.020	0.992	1.008
											Self-blame	−0.604	0.117	−0.481	−5.180	<0.001	0.992	1.008
3	17.056	3	81	<0.001	0.387	0.089	11.723	1	81	0.001	Constant	25.382	2.033		12.484	<0.001		
											Diagnosis	2.126	0.897	0.207	2.370	0.020	0.990	1.010
											Self-blame	−0.477	0.116	−0.380	−4.128	<0.001	0.891	1.122
											Catastrophizing	−0.451	0.132	−0.315	−3.424	0.001	0.894	1.119
4	16.457	4	80	<0.001	0.451	0.064	9.372	1	80	0.003	Constant	21.771	2.267		9.604	<0.001		
											Diagnosis	1.207	0.905	0.118	1.333	0.186	0.881	1.135
											Self-blame	−0.498	0.110	−0.397	−4.517	<0.001	0.888	1.126
											Catastrophizing	−0.368	0.128	−0.257	−2.868	0.005	0.854	1.171
											Positive reappraisal	0.364	0.119	0.275	3.061	0.003	0.848	1.179
5	15.404	5	79	<0.001	0.494	0.042	6.592	1	79	0.012	Constant	23.665	2.312		10.234	<0.001		
											Diagnosis	1.502	0.883	0.146	1.702	0.093	0.866	1.154
											Self-blame	−0.426	0.110	−0.339	−3.858	<0.001	0.829	1.206
											Catastrophizing	−0.350	0.124	−0.245	−2.819	0.006	0.851	1.175
											Positive reappraisal	0.352	0.115	0.266	3.060	0.003	0.847	1.181
											Self-blame parent	−0.331	0.129	−0.216	−2.567	0.012	0.905	1.105

**Table 5:** Hierarchical Linear Regression Analysis with ILC proxy as Dependent Variable.

Model	*F*	df1	df2	*p*	R^2^	Change Statistics					Variable Added	B	Std. Error	β	*t*	*p*	Collinearity Statistics	
						R^2^	*F*	df1	df2	*p*							TOL	VIF
1	6.328	1	83	0.014	0.071	0.071	6.328	1	83	0.014	Constant	17.756	1.393		12.751	<0.001	1.000	1.000
											Diagnosis	2.177	0.866	0.266	2.516	0.014		
2	14.771	2	82	<0.001	0.265	0.194	21.640	1	82	<0.001	Constant	24.195	1.862		12.991	<0.001		
											Diagnosis	1.690	0.782	0.207	2.162	0.034	0.982	1.018
											Rumination (parent)	-0.559	0.120	−0.444	−4.652	<0.001	0.982	1.018
3	11.235	3	81	<0.001	0.294	0.029	3.325	1	81	0.072	Constant	24.531	1.846		13.290	<0.001		
											Diagnosis	1.986	0.788	0.243	2.521	0.014	0.940	1.063
											Rumination (parent)	−0.401	0.147	−0.319	−2.737	0.008	0.641	1.560
											Self-blame (parent)	−0.258	0.142	−0.212	−1.823	0.072	0.647	1.545
4	9.809	4	80	<0.001	0.329	0.035	4.201	1	80	0.044	Constant	21.421	2.362		9.068	<0.001		
											Diagnosis	1.964	0.773	0.240	2.543	0.013	0.940	1.064
											Rumination (parent)	−0.338	0.147	−0.269	−2.297	0.024	0.613	1.632
											Self-blame (parent)	−0.238	0.139	−0.195	−1.709	0.091	0.644	1.553
											Positive refocusing (parent)	0.212	0.103	0.198	2.050	0.044	0.902	1.108

## Data Availability

The data presented in this study are available on request from the corresponding author.

## Appendix A

**Table A1:** Difference between adolescents diagnosed with IBD and T1DM in the duration of illness.

	IBD		T1DM				
	*M*	*SD*	*M*	*SD*	*t* (80.99)	*p*	Cohen’s *d*
Duration of illness	3.56	3.19	7.01	4.23	−4.27	<0.001	0.92

Note: IBD = inflammatory bowel disease, T1DM = type-1 diabetes, ILC = Inventory of Quality of Life.

**Table A2:** The results of Mann-Whitney U tests comparing the adolescent and parental age between the IBD and T1DM groups.

	IBD	T1DM		
	Median	Median	U	*p*
Adolescent age	16.00	15.00	671.500	0.04
Parental age	46.00	45.00	801.500	0.48

Note: *p* < 0.025 (α = 0.05/2, using Bonferroni correction), IBD = inflammatory bowel disease, T1DM = type-1 diabetes, ILC = Inventory of Quality of Life.

**Table A3:** The results of chi-square tests between the diagnosis and demographic characteristics.

	*X* ^2^	df	*p*		IBD	T1DM	Total
Adolescent sex	1.163	1	0.281	Male	16	13	29
				Female	24	32	56
Parent sex	0.580	1	0.446	Male	4	7	11
				Female	36	38	74
Place of residence	3.327	2	0.189	Capital city	13	10	23
				City	17	28	45
				Village	10	7	17
Highest education of the parent	0.076	2	0.963	College +	6	6	12
				High school	9	11	20
				Less than high school	25	28	53
Family	2.965	2	0.227	Dual parent	32	36	68
				Single parent	5	9	14
				Other	2	0	2

Note: *p* < 0.01 (α = 0.05/5, using Bonferroni correction), IBD = inflammatory bowel disease, T1DM = type-1 diabetes, ILC = Inventory of Quality of Life, College + = The highest level of education attained by any parents is college degree or higher.

**Table A4:** Descriptive statistics and reliabilities of study variables in each diagnostic group.

	IBD							T1DM						
	M	SD	Min	Max	Skewness	Kurtosis	Cronbach’s α	M	SD	Min	Max	Skewness	Kurtosis	Cronbach’s α
ILC	18.74	4.77	8.00	28.00	−0.01	−0.40	0.77	21.44	5.20	8.00	28.00	−0.65	−0.27	0.86
ILC proxy	19.93	3.97	13.00	26.00	−0.13	−1.30	0.81	22.11	4.00	13.00	28.00	−0.51	−0.26	0.79
CERQ														
Self-blame	1.35	4.70	4.00	2.00	0.50	−0.73	0.91	9.62	3.51	4.00	18.00	0.69	0.03	0.79
Acceptance	12.26	3.41	6.00	19.00	0.00	−0.88	0.57	13.84	3.22	6.00	2.00	0.05	−0.17	0.65
Rumination	1.08	4.69	4.00	19.00	0.37	−0.97	0.88	1.37	3.85	4.00	2.00	0.52	0.03	0.72
Positive refocusing	1.37	4.30	4.00	19.00	0.27	−0.83	0.89	12.02	4.32	4.00	2.00	0.07	−1.04	0.87
Planning	12.59	3.70	4.00	2.00	−0.27	0.22	0.73	13.86	3.45	7.00	19.00	−0.33	−1.02	0.75
Positive reappraisal	11.11	3.37	4.00	18.00	−0.16	−0.01	0.65	13.71	3.96	6.00	2.00	−0.16	−1.02	0.83
Putting into perspective	11.17	3.88	5.00	19.00	0.25	−0.92	0.80	13.02	4.37	4.00	2.00	−0.10	−0.77	0.86
Catastrophizing	8.49	3.69	4.00	17.00	0.66	−0.39	0.75	8.00	3.55	4.00	15.00	0.80	−0.45	0.78
Other-blame	7.03	3.38	4.00	17.00	1.13	0.73	0.86	7.27	3.14	4.00	15.00	0.99	0.39	0.87
CERQ proxy														
Self-blame	8.95	2.78	4.00	17.00	0.72	0.71	0.68	9.56	3.82	4.00	19.00	0.69	−0.02	0.88
Acceptance	12.53	3.00	6.00	19.00	−0.15	0.33	0.59	13.23	3.25	8.00	2.00	0.12	−0.70	0.66
Rumination	1.65	3.45	5.00	19.00	0.58	−0.20	0.76	9.78	3.08	4.00	17.00	−0.20	−0.06	0.63
Positive refocusing	1.74	3.79	4.00	2.00	0.39	−0.62	0.88	11.04	3.90	5.00	2.00	0.48	−0.40	0.91
Planning	15.03	3.32	4.00	2.00	−0.98	2.10	0.77	14.90	3.53	7.00	2.00	−0.28	−0.62	0.80
Positive reappraisal	12.93	3.42	7.00	18.00	−0.43	−0.60	0.67	13.44	4.05	5.00	2.00	−0.12	−0.66	0.86
Putting into perspective	11.63	3.81	4.00	2.00	0.34	−0.44	0.76	12.39	4.09	4.00	2.00	0.14	−0.36	0.84
Catastrophizing	7.35	2.60	4.00	13.00	0.48	−0.79	0.63	7.03	2.57	4.00	13.00	0.52	−0.74	0.60
Other-blame	5.78	1.98	4.00	13.00	1.50	2.99	0.84	5.60	1.81	4.00	9.00	0.52	−1.41	0.59

Note: *n* = 85. ILC = Inventory of Quality of Life, CERQ = Cognitive Emotion Regulation Questionnaire.

**Table A5:** Differences between adolescents diagnosed with IBD and T1DM in quality of life and cognitive emotion regulation.

	IBD		T1DM				
	*M*	*SD*	*M*	*SD*	*t*(83)	*p*	Cohen’s *d*
ILC	18.7436	4.76562	21.4378	5.20349	−2.478	0.015	0.539
ILC proxy	19.9337	3.96925	22.1111	3.99558	−2.516	0.014	0.546
Self-blame	10.3472	4.70336	9.6222	3.51160	0.811	0.420	0.217
Acceptance	12.2635	3.41189	13.8444	3.21895	−2.197	0.031	0.477
Rumination	10.0792	4.69414	10.3712	3.85448	−0.315	0.754	0.068
Positive refocusing	10.3679	4.30285	12.0222	4.32482	−1.764	0.081	0.383
Planning	12.5887	3.70149	13.8629	3.45261	−1.642	0.104	0.356
Positive reappraisal	11.1131	3.37147	13.7111	3.96360	−3.234 *	0.002	0.706
Putting into perspective	11.1707	3.88220	13.0193	4.36876	−2.051	0.043	0.447
Catastrophizing	8.4923	3.69106	8.0000	3.54837	0.627	0.533	0.136
Other-blame	0.8037	0.19225	0.8249	0.17809	−0.528	0.599	0.114
Self-blame parent	8.9500	2.78227	9.5633	3.82431	−0.836	0.405	0.183
Acceptance parent	12.5250	2.99562	13.2298	3.24668	−1.036	0.303	0.226
Rumination parent	10.6500	3.44592	9.7779	3.08011	1.232	0.221	0.227
Positive refocusing parent	10.7366	3.79178	11.0394	3.90313	−0.362	0.718	0.079
Planning parent	15.0250	3.32425	14.9016	3.53167	0.165	0.869	0.036
Positive reappraisal parent	12.9250	3.42231	13.4390	4.04828	−0.628	0.532	0.137
Putting into perspective parent	11.6250	3.81419	12.3903	4.08529	−0.889	0.376	0.194
Catastrophizing parent	7.3500	2.59734	7.0288	2.57166	0.572	0.569	0.124
Other-blame parent	0.7403	0.13302	0.7271	0.13587	0.453	0.652	0.098

Note. * *p* < 0.0025 (α = 0.05/20, using Bonferroni correction), IBD = inflammatory bowel disease, T1DM = type-1 diabetes, ILC = Inventory of Quality of Life.
